# Ethical and technical considerations for the creation of cell lines in the head & neck and tissue harvesting for research and drug development (Part I): Techniques of tissue harvesting and propagation

**DOI:** 10.1186/1755-7682-2-8

**Published:** 2009-04-03

**Authors:** Tahwinder Upile, Waseem Jerjes, Panagiotis Kafas, Sandeep U Singh, Holger Sudhoff, Jaspal Mahil, Ann Sandison, Colin Hopper

**Affiliations:** 1 Head & Neck Centre, University College London Hospital, London, UK; 2 Head and Neck Department, Charing Cross Hospital, London, UK; 3 Department of Surgery, University College London Medical School, London, UK; 4 Department of Oral Surgery and Radiology, School of Dentistry, Aristotle University, Thessaloniki, Greece; 5 Department of Otolaryngology, Bielefeld University, Bielefeld, Germany; 6 Department of Histopathology, Imperial College & Charing Cross Hospital, London, UK; 7 The Royal National Throat, Nose and Ear Hospital, 330/332 Grays' Inn Road, London, WC1X 8EE, UK

## Abstract

**Background:**

Although much has been published for the development of cell lines, these were lab based and developed for scientific technical staff.

**Objective of review:**

We present a simple and successful protocol for the development of cell lines and tissue harvesting for the clinical scientist. We also discuss the ethical implications of tissue retention and present a generic consent form.

**Conclusion:**

The advantages of hospital-based cell line creation are numerous. We can be more certain that cell lines are developed from the particular tissues of interest and accurate anatomical and appropriate clinico-pathological control tissues are also harvested. We can also be certain of less cell line cross contamination.

## Background

In this molecular diagnostic age, we have a duty to our patients to try to advance and improve treatment. One of the main areas of research nowadays is related mainly to cell cultures and their applications increases everyday [1-7].

Human cells will usually continue to grow if supplied with the appropriate nutrients and conditions. Cell culture or cell lines helps us to investigate the physiology and biochemistry of the cell (i.e. cell metabolism) and to test the effect of various chemicals or drugs on specific cell types, i.e. *in vitro *assays of the effect of chemotherapy, radiotherapy and gene therapy regimes to examine the possibility for resistance to optimise treatment. This procedure is very similar to microbiological sensitivities to assess bacterial susceptibility to antibiotics. Furthermore tissue or pathological samples taken at operation can be tested against protein chips or have their genetic material extracted and run against gene chips. This may provide direct prognostic information as to the likely clinical progression and response of the pathological process [1-7].

Cell lines have been used in generating artificial tissues (tissue engineering), i.e. artificial skin, and to synthesize valuable biological compounds from large scale cell cultures, i.e. therapeutic proteins. One of the main advantages of cell lines is the consistency and reproducibility of results; however, cell characteristics can change after a period of continuous growth. Cells are able to adapt to different culture environments by varying the activities of their enzymes [1-3]. A realisation of the cell's microenvironment is fundamental to the successful creation of cell lines. For instance exposure of the cell culture to air allows the mixed cell culture to undergo cell mediated separation into overlying epidermal cells and underlying fibroblasts without significant chemical or physical alteration that may change cellular behaviour (expression or multiplication).

Although much has been published for the development of cell lines [1-7], these were lab based and developed for scientific technical staff. We, however, present a simple and successful protocol for the development of cell lines and tissue harvesting for the clinical scientist. These techniques do not require high technology and can be performed by most clinicians in most hospitals; this will usually require basic knowledge of cell culture concepts (Table 1) and the materials used.

**Table 1:** Concepts in cell culture

Isolation of cells	Cells can be isolated from tissues for *ex vivo *culture in several ways (purified from blood or by enzymatic digestion)
Maintaining cells in culture	Cells are grown and maintained at an appropriate temperature, gas mixture and growth media (vary in pH, glucose concentration, growth factors, and the presence of other nutrient components) in a cell incubator. Some times extracellular matrix components (i.e. collagen or fibronectin) are needed to increase its adhesion
Manipulation of cultured cells	Cells generally continue to divide in culture, this usually lead to nutrient depletion in the growth medium, Accumulation of apoptotic/necrotic cells, cell cycle arrest or promiscuous and unwanted cellular differentiation due to cell-to-cell contact. To avoid these problems cultured cells is manipulated. Most common manipulation: media changes, passaging cells, and transfecting cells
Media changes	To replenish nutrients and avoid the build up of potentially harmful metabolic byproducts and dead cells by centrifugation or aspiration
Passaging (splitting) cells	Involves transferring a small number of cells into a new vessel. This can either be done by introducing a small amount of culture containing a few cells diluted in a larger volume of fresh media or by a mixture of trypsin-EDTA, however other enzyme mixes are now available for this purpose
Transfection and transduction	Involves the introduction of foreign DNA and the cells will express a protein of interest. More recently, the transfection of RNAi constructs have been realised as a convenient mechanism for suppressing the expression of a particular gene/protein

The head and neck contains the most diverse range of accessible histopathological entities. Tissues taken are not just tumour cell lines but mucosa and cartilage (used later for tissue engineering). Little to date has been published in the literature with regard to harvesting this potentially wasted resource. We also discuss the ethics implications of tissue retention and present a generic consent form, which maybe adapted to suit individual institutions (see Part II).

## Methods

### Creation of Cell Lines

The creation of cell lines is an art, which develops with practice and the adaptation of local resources to facilitate tissue growth.

Primary cultures represent (heterogeneous but still closely represent the parent cell types) freshly isolated cultures until sub-cultured. Several sub-cultures (passages) onto fresh media cause the cell lines either to transform (become continuous) or die. Sub-cultured cell lines may be different in morphology and have slight chromosomal variation when compared to the primary cultures. Cell lines grow attached to a solid surface but can also grow in an unattached suspension culture in some cases. The substrate used may affect cell behaviour.

The basic materials for the creation of cell lines include: chemicals, incubators (humidified incubators 37°C – 5% CO_2 _and 95% air), snap freezing materials (liquid nitrogen), tissue culture and some instruments (size 15 blade scalpel and forceps) (Tables 1 and 2).

**Table 2:** Most common chemicals used in the creation of cell lines

maintenance and growth media	Dulbecco's modified Eagles medium (DMEM)-with 2 mM L-glutamine, 100 units/ml
	Penicillin and 100 ug/ml streptomycin, 2.5 ug/ml
	Amphotericin B (An antimicrobial cocktail to reduce chance of infection)
	20% of Fetal Calf Serum- 10% v/v-decomplemented at 56'C for 45 min, (A growth medium containing several important growth factors)
	Phosphate buffer saline (for isotonic washes and mechanical reduction in microbial load)
	7% Dimethylsulfoxide (DMSO for cryo-preservation)
	93% Fetal Calf Serum
separation of cells explants for cell lines	Trypsin 0.05%, 0.01% bovine pancreatic trypsin (to allow detachment of cells from underlying substrate- plastic or collagen)
	Na_2_EDTA
	Defined keratinocyte serum free medium

Chemicals are usually used for maintenance, as growth media and for separation of cells explants for cell lines (Table 2) and these procedures takes place in various universal tubes (Appendix 1).

### Ethics and consent

Research and ethical approval is ideally obtained with regards the consent and subsequent tissue use. The voluntary nature of the process must be emphasized and no form of duress implied, ideally the process is carried out well ahead of any procedure. We present our current consent form for modification and usage (see Part II).

Prospective patient data is entered on a proforma or directly into a database detailing i.e. family history, carcinogenic exposure, TNM stage (with volumetric staging), previous and proposed treatment and duration with later entry of prognostic, morbidity and mortality data. A note is made of the anonimised patient sample number. These anonimised records are held in a secure computer and written form.

### Site and sampling

Controls may be selected on the basis of anatomy and/or exposure to carcinogens or previous treatment fields, i.e. in the head & neck region contralateral piriform fossa, non affected portion of tissues. Control tissue can be taken at fixed distances from the pathology edge, i.e.1, 2, 5 cm for assessment of field effect or suppressed potential or dormant pathology clones. This is carried out with the auspices of institutional ethics committee, research and development department and fully informed patient consent.

Each head & neck pathology must be taken into context, i.e. assessing the exposure to potentially contaminated body fluids, i.e. saliva, pus necessitating antimicrobial treatment or hypoxic conditions affecting viability and cell line growth.

A sample for histopathological confirmation of tissue type (pathology type, grade, histology of control tissue) is also always taken. Furthermore, confirmation of infection by microbiological culture and later confirmation of irradication of infection (i.e. by myoplasma PCR [polymerase chain reaction]) can be performed in house by the local microbiological laboratory.

For non mucosal non exposed head and neck pathologies, one hopes to sample non necrotic tissue. Here the risk of contamination is reduced but hypoxic factors may affect the pathological cell viability.

For mucosal (aero-digestive)/skin pathology with exposure to contamination, i.e. salivary content or skin flora, the risks of infection (i.e. mycoplasma) which may affect tissue growth and cellular response necessitating the use of appropriate antibiotics, antifungals or antivirals.

### Tissue processing

Processing is carried out in a sterile laminar airflow environment as is found in the typical operating theatre environment.

Tissue can be snap frozen within seconds of excision by full sample immersion in liquid nitrogen. Alternatively tissue may be placed in RNA'ase later (to prevent RNA loss) for later processing. Separate forceps and scalpel are used for primary, secondary, metastases and control tissue to reduce cross contamination of genetic material.

### The development of cell lines from tissue explants

Pathological tissue is resected at the time of major surgery and material surplus to pathological diagnostic requirements is immediately divided into three parts. The first part (for DNA, RNA and protein extraction) is immersed in liquid nitrogen and the second part (for later immunohistochemical and *in situ *hybridisation studies) is frozen on dry ice and both are stored at -80°C. The third part is used for the establishment of cell lines according to the methodology described with monitor modifications.

## Results

### Collection of tissue materials and establishment of new cell lines

A tissue sample, where material in excess of diagnostic requirements is available, is obtained from patients undergoing major surgical resection with curative or palliative intent. The establishment of pathological tissue cell lines is carried out as previously described with some modifications (Tables 1 and 2).

Biopsy material is soaked briefly in absolute alcohol (2–3 seconds) and then washed twice in DMEM containing 200 iu/ml penicillin, 200 ug/ml streptomycin and 5 ug/ml fungizone (This is in an attempt to prevent bacterial and fungal infections contaminating the cell lines).

Surgical specimens are further processed in cold "establishment media" consisting of DMEM/20% FCS with 2.5 ug/ml Amphotericin-B (Antifungal agent). Tissues are reduced to 0.5–1 mm^3 ^fragments with crossed size 20 scalpel blades under aseptic conditions (this creates smaller potential tissue explants which have a larger surface area to volume ratio allowing the diffusion of nutrients, oxygen and wastes increasing the likelihood of growth of cells from the explant). After three washes with PBS containing 2.5 ug/ml Amphotericin-B, about 8–10 fragments are transferred either to:

a) A 25-cm^2 ^plastic culture flask containing not more than 3 ml of "establishment media". Cultures are maintained in humidified incubators at 37°C in an atmosphere of 5% CO_2 _and 95% air for at least four days without disturbance. The medium is renewed twice weekly once the tissue fragments had become firmly attached. Within 7–10 days, migration of epithelial and fibroblast cells from the pathological explants should be apparent.

b) An incubation mixture of 5 ml of trypsin type III (SIGMA) containing 100 iu/ml penicillin, 100 ug/ml streptomycin, and 2.5 ug/ml fungizone initially at 4°C for 12 hours and then at 37°C for 30 minutes. The digested tissues are then resuspended in complete medium (DMEM containing 20% FCS, 100 iu/ml penicillin, 100 ug/ml streptomycin, 2.5 ug/ml fungizone, 0.075% additional sodium bicarbonate, 0.6 mg/ml additional L-glutamine and 0.5 ug/ml hydrocortisone) and seeded into 60 mm tissue culture Petri dishes.

All cultures are incubated in a humidified of 5% CO_2_/95% air at 37°C air and the medium is changed twice weekly. In later culture passages, cell lines are grown in DMEM containing 10% FCS and free of all antibiotics (this in an attempt to encourage the cell line to revert back to a more 'natural' mode of behaviour not influenced by the chemical properties of the antibiotics).

Fibroblast outgrowth is controlled by selective detachment via mechanical removal using a cell-scraper or exposure to 0.05% trypsin/0.04% Na_2_EDTA (this allows preferential detachment of selected cells improving the purity of the remaining cells). After the first passage, cultures are maintained in DMEM/10% FCS without anti-fungal agent. Cells then are subcultured at 95% confluence with a dilution of 1:6 to 1:8 and are stored frozen at -135°C.

New cell lines are used during passages 5–15, whereas fibroblasts and normal keratinocytes are used at passages 3 through 5. Human keratinocytes are established from primary cultures of normal oral mucosa and maintained in defined keratinocyte-serum free medium (SIGMA), (as their maintenance growth factor cf FCS).

### Subculturing, maintenance and expansion of established cell cultures

All cell lines are cultured routinely at 37°C in a humidified atmosphere of 5% CO_2 _and 95% air. Cells are maintained in monolayer culture and passaged weekly by incubation with 0.01% bovine pancreatic trypsin in PBS containing 0.04% Na_2_EDTA for about 3–5 min at 37°C. Detached cells were collected in DMEM/10% FCS, mixed into a single-cell suspension in a universal and pelleted by centrifugation in an IEC Centra-7 centrifuge for 3 minutes at 800 g. Following resuspension in fresh, pre-warmed medium, cell counts and viability estimation are carried out by making a dilution of cell suspension in trypan blue and counting using a haemocytometer counting chamber. Cells are diluted to the required density (1–5 × 10^4^/ml) before plating into culture flasks (5 ml/25 cm^2^, 25 ml/80 cm2, 50 ml/170 cm^2^) or plates (200 ml/well of 96-well plates, 2 ml/well of 24-well plates, 5 ml/well of 6-well plates). Medium is replaced two or three times per week. Vigilance is kept for signs of microbial infection (bacterial or fungal), any cell aliquots that are infected should be discarded and all apparatus sterilised.

### Long term storage of cells

Cells of early passages and high viability (>80%) are stored at -135°C in liquid nitrogen (this slows their deterioration and allows later resurrection and specific experimental usuage making the process overall more efficient). Between 10^6^-10^7 ^cells are pelleted by centrifugation and the supernatant is discarded. The cells are resuspended in 1 ml of an ice-cold freezing mixture containing 7% DMSO and 93% FCS and transferred to 1.5 ml cryo-tubes. After controlled freezing, the samples are immersed in liquid nitrogen. When required, aliquots of cells are thawed quickly at 37°C, diluted into 20 ml DMEM/10% FCS in a 30 ml universal tube. After centrifugation, the cells are washed once more with 20 ml of DMEM/10% FCS and then plated out into 25 cm^2 ^flasks. Liquid nitrogen storage enables one to maintain a large number of cell line aliquots of low passage number without the need to immortalise the cell lines by transfections which may change their behaviour further away from that of the pathology of origin. By doing this a large number of experiments can be efficiently carried out with minimal loss of the 'natural pathological cell' behaviour.

### Sample storage

Tissue is stored in cryotubes in liquid nitrogen containers. For material refrigerated at -80°C a back up power supply and fail safe is essential. In either case the shelf position or row is noted as is the numbered cryotube label with an encryption code based on an algorithm that codes for the unique patient identity. These anonimised records are held in a secure computer and written form. It is essential data is collected prospectively.

### Head & neck cell lines

**Figure 1:**
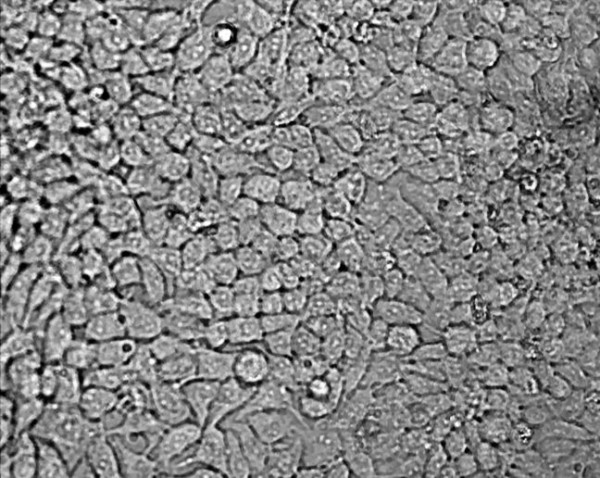
**Morphological examination by phase contrast micrography in the late log phase of growth (original magnification ×200)-HN1A oral primary (on plastic)**.

**Figure 2:**
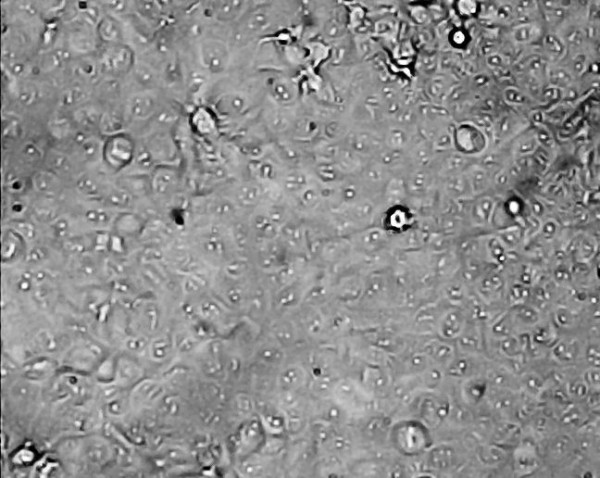
**Morphological examination by phase contrast micrography in the late log phase of growth (original magnification ×200)-HN1B metastasis (on plastic)**.

**Figure 3:**
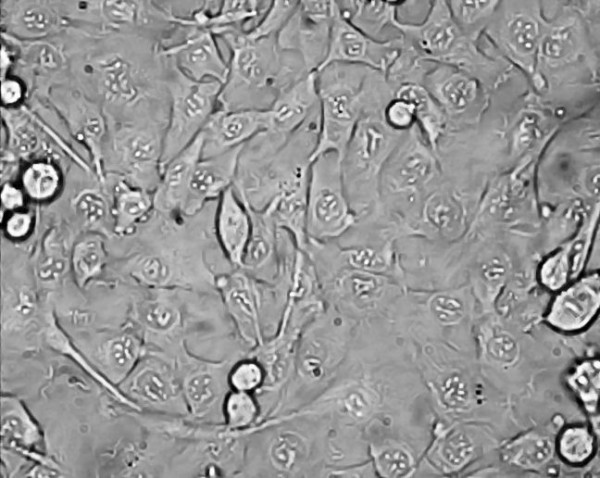
**Morphological examination by phase contrast micrography in the late log phase of growth (original magnification ×200)-HN2A oropharyngeal primary (on plastic)**.

**Figure 4:**
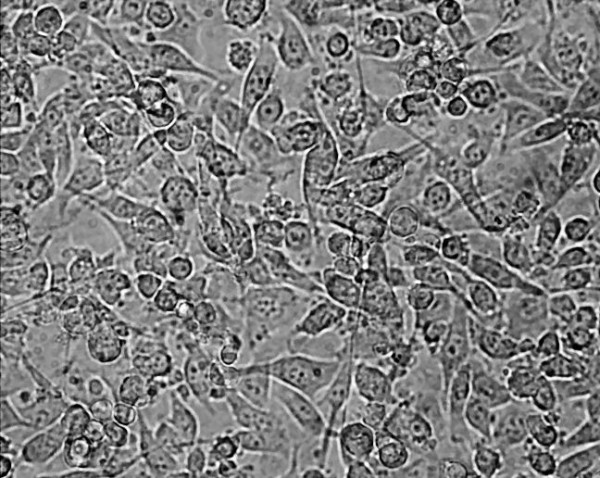
**Morphological examination by phase contrast micrography in the late log phase of growth (original magnification ×200)-HN2B metastasis (on plastic)**.

**Figure 5:**
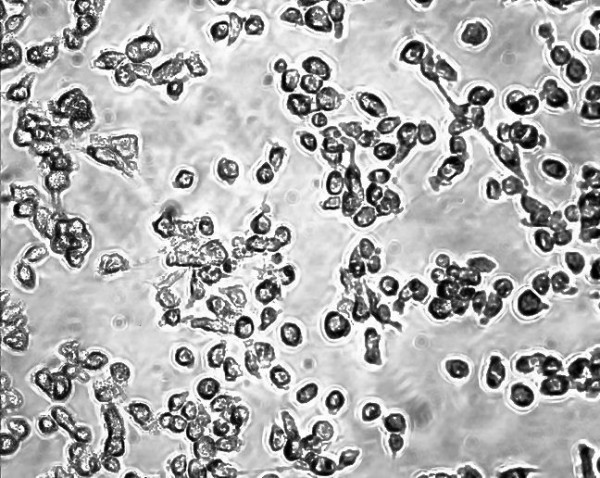
**Morphological examination by phase contrast micrography in the late log phase of growth (original magnification ×200)-HN2A oropharyngeal primary (on collagen)**.

**Figure 6:**
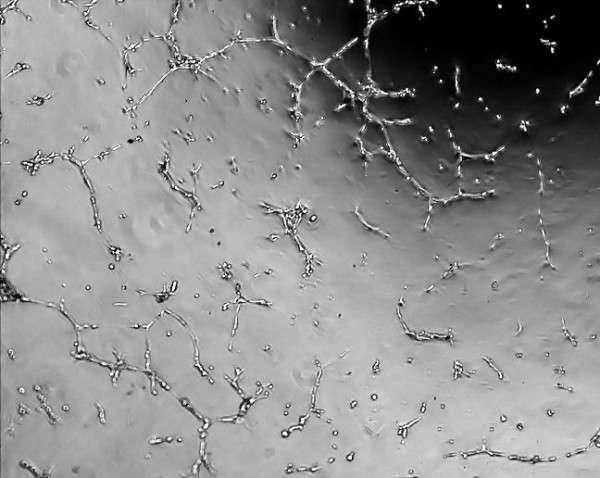
**Morphological examination by phase contrast micrography in the late log phase of growth (original magnification ×200)-HN2B metastasis (on collagen)**.

An example of some head and neck cell lines developed using this protocol. HN1a represents primary oral tumour whilst HN1b represents a cell lines derived from a lymphatic metastasis. HN2 represents a similar primary oropharyngeal tumour (A) and secondary (B) cell line; we also illustrate the change in morphology of the cell depending on substrate (plastic and collagen). **Note the changed growth pattern in the metastatic cell lines **(Figures 1, 2, 3, 4, 5 and 6).

Initially we were successful in 30% of cases but as experience grew, we developed cell lines in over 70% of cases. The main difficulties tended to be infection (resolved with strict sampling testing and cleaning of cell lines) and lack of viability (resolved by sampling away from areas of tumour necrosis). Fibroblast contamination was control by differential washes and mechanical detachment.

## Discussion & Conclusion

We present a simple and successful protocol for the development of cell lines and tissue harvesting. It does not require high technology and can be performed by most clinical scientists in most hospitals.

The advantages of hospital based cell line creation are numerous. We can be more certain that cell lines are developed from the particular tissues of interest and accurate anatomical and appropriate clinico-pathological control tissues are also harvested. We can also be certain of less cell line cross contamination i.e. laboratory HELA cells contamination of nearly over a half of all existing cell lines which has put much previous fundamental basic science research into question.

Modifications to this may include the use of autologous donor serum instead of the standard fetal calf serum. Antibody depleted heterologous serum may even be employed. Cells may be grown on type IV collagen or irradiated fibroblasts rather than plastic to better the 'in-vivo' microenvironment of the cells. This will affect its cyto-skeletal messenger system and eventually gene expression and protein synthesis. Finally, as more is realized regarding cell interdependency co-culture can be contemplated.

Essential to success is the multidisciplinary approach with particular regards to the pathologist. Nothing should prevent accurate pathological diagnosis and we prefer all samples to be taken in full cooperation with the specialist surgical pathologist.

Consent is also more proximate and assurance can be given of appropriate usage.

## Abbreviations

BSA: Bovine serum albumin; DMEM: Dulbecco's modified Eagle's medium; DMSO: Dimethylsulfoxide; DWW: Double distilled water; ECM: Extracellular matrix; ELISA: Enzyme-linked immunosorbent assay; FCS: Foetal calf serum; HNSCC: Head and neck squamous cell carcinoma; mAb: Monoclonal antibody; min: Minute; mw: Molecular weight; NRG: Neuregulin; OD: Optical density; pAb: Polyclonal antibody; PBS: Phosphate buffered saline; PBSAz: Phosphate buffered saline containing 0.2% NaN_3_; sc: Subcutaneous; SDS: Sodium dodecyl sulphate; TBS: Tris-buffered saline.

## Competing interests

The authors declare that they have no competing interests.

## Authors' contributions

TU, WJ, PK, SUS, HS, JM, AS and CH contributed to conception and design, carried out the manuscript editing and manuscript review. All authors read and approved the final manuscript.

## Appendices

Appendix 1: Most common universal tubes used in the creation of cell lines

Culture flasks (5 ml/25 cm^2^, 25 ml/80 cm^2^, 50 ml/170 cm^2^)

Culture plates (200 ul/ml of 96 well plate, 2 ml/well of 24 plates, 5 ml/wel of 6 well plates)

1.5 ml cryotubes

30 ml universal tubes

25 cm^2 ^plastic culture flasks

The flask caps allow exchange of gases with a 5% CO_2 _enhancement
